# Antioxidative Dietary Compounds Modulate Gene Expression Associated with Apoptosis, DNA Repair, Inhibition of Cell Proliferation and Migration

**DOI:** 10.3390/ijms150916226

**Published:** 2014-09-15

**Authors:** Likui Wang, Shijuan Gao, Wei Jiang, Cheng Luo, Maonian Xu, Lars Bohlin, Markus Rosendahl, Wenlin Huang

**Affiliations:** 1 CAS Key Laboratory of Pathogenic Microbiology and Immunology, Institute of Microbiology, Chinese Academy of Sciences, Beijing 100101, China; E-Mails: gao_shijuan@163.com (S.G.); jiangw@im.ac.cn (W.J.); 2 Department of Biotechnology, University of Tartu, Tartu 51010, Estonia; E-Mail: luo58@yahoo.com; 3 Mysenso Oy, Hollola 15870, Finland; E-Mail: markus.rosendahl@gmail.com; 4 Department of Food and Environmental Sciences, Division of Food Chemistry, University of Helsinki, Helsinki F-00014, Finland; E-Mail: xumaonian@gmail.com; 5 Division of Pharmacognosy, Department of Medicinal Chemistry, Biomedical Center University of Uppsala, Uppsala 75123, Sweden; E-Mail: lars.bohlin@fkog.uu.se; 6 State Key Laboratory of Oncology in South China, Cancer Center, Sun Yat-Sen University, Guangzhou 510060, China

**Keywords:** anti-inflammatory, antioxidants, apoptosis, cell migration and invasion, DNA repair

## Abstract

Many dietary compounds are known to have health benefits owing to their antioxidative and anti-inflammatory properties. To determine the molecular mechanism of these food-derived compounds, we analyzed their effect on various genes related to cell apoptosis, DNA damage and repair, oxidation and inflammation using* in vitro* cell culture assays. This review further tests the hypothesis proposed previously that downstream products of COX-2 (cyclooxygenase-2) called electrophilic oxo-derivatives induce antioxidant responsive elements (ARE), which leads to cell proliferation under antioxidative conditions. Our findings support this hypothesis and show that cell proliferation was inhibited when COX-2 was down-regulated by polyphenols and polysaccharides. Flattened macrophage morphology was also observed following the induction of cytokine production by polysaccharides extracted from viili, a traditional Nordic fermented dairy product. *Coix lacryma-jobi* (coix) polysaccharides were found to reduce mitochondrial membrane potential and induce caspase-3- and 9-mediated apoptosis. In contrast, polyphenols from blueberries were involved in the ultraviolet-activated p53/Gadd45/MDM2 DNA repair system by restoring the cell membrane potential. Inhibition of hypoxia-inducible factor-1 by saponin extracts of ginsenoside (Ginsen) and Gynostemma and inhibition of S100A4 by coix polysaccharides inhibited cancer cell migration and invasion. These observations suggest that antioxidants and changes in cell membrane potential are the major driving forces that transfer signals through the cell membrane into the cytosol and nucleus, triggering gene expression, changes in cell proliferation and the induction of apoptosis or DNA repair.

## 1. Introduction

Oxidative stress and inflammation are common features of many chronic diseases and their complications, and have also been linked to aging and carcinogenesis. Previous studies have shown that approximately one third of all known plants and vegetables have antioxidant and anti-inflammatory properties and consumption of a healthy diet rich in these is thought to help counter disease processes.

It is commonly regarded that non-receptor mediated regulation of cells by dietary compounds is negligible. However, recent findings show that dietary compounds may cause a switch in receptor-regulated signaling pathways and impairment of these pathways is known to be associated with type 2 diabetes and other metabolic diseases. Studies have shown that the downstream products of cyclooxygenase-2 (COX-2) control one of the most important internal antioxidant pathways: the nuclear factor (erythroid-derived 2)-like 2 (Nrf2)/Kelch-like ECH-associated protein 1 (Keap1)/antioxidant responsive element (ARE) pathway [1].

Research in China has recently focused on medicinal food by pharmacologically validating established dietary herbal compounds and optimizing their function through combinations or processing based on traditional practices. This review describes some of the antioxidative dietary compounds we have studied and analyzes our experimental results involving the role of polysaccharides in inducing apoptosis. The association of antioxidants with the COX-2/Nrf2/ARE pathway, the antioxidant effects of polyphenols on P53/Gadd45/MDM2 gene expression and its association with DNA repair, as well as the role of hypoxia-inducible factor (HIF)-1 and S100A4 genes in the migration and invasion of cancer cells are also discussed.

## 2. Coix Polysaccharides Induce Cancer Cell Apoptosis

The ancient food crop coix (*Coix lachryma-jobi* L.; also known as adlay seed) is widely cultivated in the warm regions of Asia, Africa and the Mediterranean Rim. The grain is prepared for eating by roasting and may be eaten dry, used for porridge or processed into flour [2]. In addition to polysaccharides, coix is rich in protein, fat, carbohydrates, amino acids, vitamins and inorganic salts. In traditional Chinese medicine coix is used as a diuretic, an anti-inflammatory drug, an anti-cancer drug, an analgesic and a nutrient [3]. Studies have shown that coix contains a large number of lipopolysaccharides, including palmitic acid, stearic acid, octadecadienoic acid, oleic acid and linoleic acid. It also contains oligosaccharides with free radical scavenging and other antioxidant properties [4]. Apirattananusorn* et al.* [5] found that non-starch polysaccharides (mainly arabinoxylans) in coix are present in the alkaline rather than the water extract, and that the arabinoxylan molecule has a (1,4)-linked-d-xylan main chain highly substituted with arabinose units.

A number of studies have reported the bioactivities of compounds isolated from coix. For example, a methanol extract of adlay seed suppressed the expression of COX-2, a key enzyme that catalyzes the transformation of arachidonic acid into prostaglandins [6]. It is known that COX-2 is expressed in human lung cancer cells and coix has shown significant anti-proliferative effects on these cells due to its inhibition of COX-2 gene expression [7]. The oil extract of adlay seed has also been shown to inhibit fatty acid synthase and is used in anti-neoplastic therapy [8], while dehulled adlay seed suppresses early events in colon carcinogenesis and reduces COX-2 protein expression [9]. In addition, coix polysaccharide was shown to possess a hypoglycemic function [10] and to improve immune system function [11].

Therefore, we have investigated the anti-cancer mechanisms of coix polysaccharides in the human lung adenocarcinoma epithelial cell line A549, by multiple methods including alkaline gel electrophoresis of single cells (comet assay) and flow cytometry. MTT (3-[4,5-dimethylthiazol-2-yl]-2,5 diphenyl tetrazolium bromide) assays showed that the polysaccharide fraction CP-1 inhibited the proliferation of A549 cells in a time- and concentration-dependent manner, with the highest inhibition observed at a CP-1 concentration of 300 µg/mL for 72 h. CP-1 induced both cell cycle arrest in S phase and apoptosis of A549 cells as demonstrated by cell cycle analysis and annexin V-fluorescein/propidium iodide staining assays [12].

The single cell gel electrophoresis assay detects DNA damage, including DNA strand breaks and alkali labile lesions, with high visual resolution [13]. The movement of DNA from the head to the tail has been described as the most obvious characteristic of apoptotic cells in the comet assay [14]. Our results showed that the length of the comet tail was proportional to the degree of DNA fragmentation. Furthermore, apoptotic cells were clearly distinguishable between control and test groups [12].

The caspase gene family plays a central role in mitochondrial-mediated apoptosis [15,16,17]. Caspase-3, which is activated by caspase-9, contributes to the execution of apoptosis via the activation, hydrolysis and proteolysis of specific substrates such as DNA-dependent protein kinases [18]. Our western blot analysis showed that CP-1 increased the expression of both caspase-3 and caspase-9, suggesting that coix polysaccharides mediate apoptosis via a caspase-dependent pathway. Furthermore, the CP-1-mediated disruption of the mitochondrial membrane potential, which typically leads to the activation of caspase-3 and caspase-9 [19], suggests that a mitochondrial-dependent pathway is involved in the induction of apoptosis by CP-1 [12].

**Figure 1:**
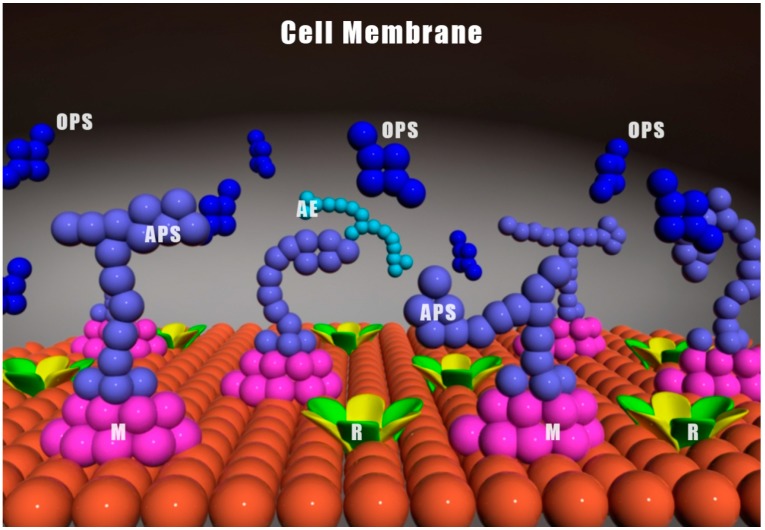
Hypothesis of how foodborne or metabolized polysaccharides/oligosaccharides interact with the cell outer membrane. **OPS**: (dropped) off polysaccharides; **APS**: attached polysaccharides; **AE**: antenna extracellular membrane polysaccharides that may absorb different wavelengths of light, changing the conformation of membrane associated proteins; **R**: receptors; **M**: membrane proteins.

To date, no relevant polysaccharide receptor has been identified on the A549 cell membrane that would enable CP-1 to interact and produce its antioxidant effects, which include interfering with cell growth, metabolism and proliferation, leading to the induction of apoptosis (Figure 1). Nevertheless, the mechanism through which CP-1 induces apoptosis in A549 cells is worthy of further investigation owing to its potential anti-tumor effects.

## 3. Inflammatory and Anti-Inflammatory Effects of Viili Polysaccharides

Viili, a semi-solid yogurt that originated in Finland, has a ropy, gelatinous consistency and a sour taste resulting from the microbial action of lactic acid bacteria (LAB) and a surface-growing fungus, *Geotrichum candidum*, which forms a velvet-like surface. Viili also contains the yeasts *Kluveromyces marxianus* and *Pichia fermentans*. Among the mesophilic LAB strains, the slime-forming LAB *Lactococcus lactis* subsp. *cremoris* produces a phosphate-containing exopolysaccharide (EPS) with a basic structure mainly composed of d-glucose, d-galactose, l-rhamnose and phosphate, with an average molecular weight of approximately 2000 kDa and a repeating unit of “→4-β-Glcp-(1→4)-β-d-Galp (1→4)-β-d-Glcp-(1→” as well as groups of -l-Rhap and -d-Galp-1-p attached to each side of Galp [20,21]. Viili has been claimed to have various functional benefits, including antioxidant, anti-inflammatory, anti-cancer and anti-aging properties, and was also reported to enhance natural immunity [22,23].

Macrophages not only constitute a principal component of the innate immune system, but they also perform pivotal roles in acute inflammatory responses and atherosclerosis [24]. Lipopolysaccharide (LPS)-stimulated macrophages can generate a variety of inflammatory mediators such as nitric oxide (NO), prostaglandin E_2_ (PGE_2_), interleukin (IL)-1β, tumor necrosis factor-α and matrix metalloprotease-9 [25]. Of these, NO is the most important molecule for inter- and intracellular signal transmission; it also mediates immune and inflammatory processes and plays a critical role in communicating physiological and pathological signals [26,27]. However, excessive levels of NO are toxic as they form free radical groups, such as superoxide (O_2_^−^), that result in production of the toxic peroxynitrite (ONOO^−^) molecule [28,29]. Therefore, changes in NO production may provide a measure to assess the effects of drugs or functional foods on the inflammatory process.

We previously studied the activity of viili exopolysaccharides (VEPS) at the cellular level, and found that they promoted the activation of macrophages in association with NO, IL-6 and IL-1β production. The release of NO and proinflammatory cytokines may activate lymphocytes, which upregulates nonspecific (innate) and specific (adaptive) immunity in humans [30]. VEPS from *L. lactis* subsp. *cremoris* were able to strongly induce NF-κB and various cytokines via two intestinal receptors associated with Peyer’s patches in swine, RP105 and MD-1 [31,32]. This inspired our investigation in mouse macrophage RAW264.7 cells. Macrophages accumulate in the small intestine and play crucial roles in both the innate and adaptive host defense against infection. They can be triggered by several different compounds, including polysaccharides and LPS. In our assay, VEPS were able to increase the proliferation of RAW264.7 cells at concentrations of 50, 100 and 200 μg/mL. VEPS also strikingly increased macrophage phagocytosis, NO secretion, iNOS gene and protein expression, the secretion and gene expression of IL-6 and IL-1β, as well as inducing a flattened morphology. Synergy was also observed when VEPS were added in the presence of LPS.

NO is recognized as a mediator and regulator of inflammatory responses. It is a nonspecific inflammatory mediator involved in triggering the proliferation of macrophages and lymphocytes, as well as stimulating the central nervous system and other immune functions* in vivo*. Of the three isoforms of NO synthase, iNOS is the most important in NO synthesis. It directly affects many physiological and pathological functions, including the relaxation of cardiovascular vessels, increasing blood flow and the promotion of cytokine emission during inflammation and at the moment of antigen recognition between T cells and antigen presenting cells [1,33,34,35]. The enhancement of NO production and gene expression by VEPS occurred in a dose-dependent manner, which strongly suggests that VEPS are immune mediators/modulators. The secretion of NO synergistically increased when both VEPS and LPS were applied, which indicates that they may share a similar mechanism. A parallel increase in iNOS was observed by both semi-quantitative RT-PCR and western blot [36].

Intestinal macrophages engulf and ingest particles to form a phagosome (or food vacuole), which in turn fuses with a lysosome to form a phagolysosome. Engulfed materials are eventually digested or degraded and either released extracellularly via exocytosis, or intracellularly to undergo further processing. The activation of intestinal macrophages by dietary factors has previously been reported [37]. After treatment with VEPS, a significant increase in macrophage phagocytosis indicates the activation of cellular functions, probably through reversible protein aggregation and motor molecular mechanisms [38]. The activation of macrophages by VEPS was also indicated by an increase in the gene expression and secretion of IL-6 and IL-1β.

It is well documented that macrophage morphology can change from round to flat when activated by LPS [39]; however, to our knowledge this is the first time that a similar morphology has been observed for RAW264.7 cells stimulated by VEPS [30]. The degree of flattening and spreading was consistent with increased proliferation, phagocytosis, iNOS expression and NO release. Although morphological alteration involves a complex, dynamic reorganization of cytoskeletal actins [40], we believe that macrophage activation by VEPS is a reversible physiological process that favorably increases proliferation of macrophages and promotes immunity. However, further studies are required to confirm this.

## 4. COX-2 (Cyclooxygenase-2) Is Involved in Anti-Inflammatory Processes via Ursolic Acid and Microbial Polysaccharides

Hepatocellular carcinoma (HCC) is estimated to be the fifth most common cause of cancer-related death worldwide [41]. Although approximately 80% of cases are reported in developing countries where the prevalence of hepatitis is high, HCC is one of very few cancers whose incidence is increasing in developed countries [42,43]. Chemotherapy has provided significant survival benefits for HCC patients, but most drugs are also associated with significant tissue toxicity. Therefore there is a need for drugs or alternative therapies that target tumor cells without compromising normal tissue function [44]. Increased concentrations of cytotoxic drugs and higher doses of radiation often fail to improve the health of patients with liver cancer, and may lead to apoptosis resistance. Therefore, an anti-cancer agent with low toxicity that preferentially induces apoptosis in human cancer cells while creating an internal oxidative environment would be very useful.

Ursolic acid (UA), a pentacyclic triterpenoid, has been identified in several vegetables and medicinal herbs [45]. UA can inhibit cell growth and induces apoptosis in some tumors through multiple pathways [46,47], including the inhibition of DNA replication, activation of caspases and down-regulation of anti-apoptotic genes [48]. UA has also been shown to specifically inhibit tumorigenesis [49], tumor progression [50], angiogenesis and tumor invasion [51]. As outlined above, the large amounts of EPS generated in viili [52] reportedly have antioxidant properties [53], which regulate immune function and lower cholesterol [54]. Moreover, the *Astragalus* species commonly used in traditional Chinese medicine, especially *A. membraneuse*, contain similar polysaccharides (*Astragalus* polysaccharides, APS) that are believed to improve or modulate immune function [55] and promote tumor cell apoptosis [56].

COX-2 is not expressed in many organs under normal physiologic conditions but is expressed in most cancer cells [57], where it is believed to inhibit cancer cell apoptosis [58] thereby causing chemotherapy resistance. Notably, COX-2 selective inhibitors have been demonstrated to inhibit tumor cell proliferation and induce apoptosis [59]. For these reasons, naturally derived COX-2 inhibitors have been investigated for use in chemotherapy and chemoprevention. We previously analyzed the synergistic effect of UA in combination with VEPS and APS on cell proliferation, morphologic changes, oxidation and COX-2 expression, and found that inhibition of cell proliferation is associated with the inhibition of COX-2 [60].

## 5. The COX-2/Nrf2/ARE Pathway

**Figure 2:**
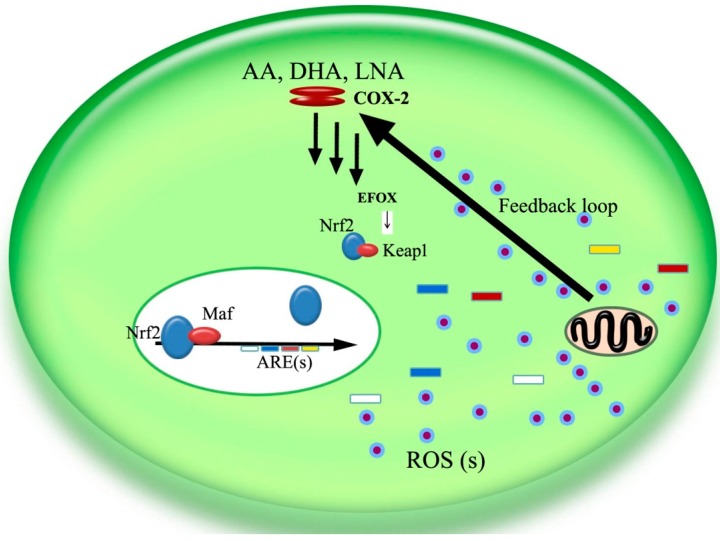
Diagram portraying a potential mechanism of COX-2’s antioxidative effect via EFOX (electrophilic oxo-derivative) molecules, where ROS(s) are hypothesized to trigger the antioxidative and anti-inflammatory effects largely based on the observation that COX-2 expression is increased in aging tissues. Abbreviations: **AA**, arachidonic acid; **DHA**, docosahexaenoic acid; **EPA**, eicosapentaenoic acid; **LNA**, linolenic acid; **ROS**, reactive oxygen species [1].

Although the development of tumors and malignancies is complex, carcinogenic events are common. Potentially cancerous cells are produced constantly, but are usually eliminated in a healthy environment. However, carcinogenesis may occur if the body’s internal antioxidant and anti-inflammatory environment changes, or if mechanisms that inhibit abnormal cell proliferation are disrupted. Recent research suggests that antioxidants and anti-inflammatories may actually increase the risk of cancer in an environment where antioxidants involved defense systems are compromised because the internal antioxidative system, the ARE genes may be induced and triggered and eventually increase cell proliferation [61,62,63,64]. However, an antioxidative environment might not curb cancer progression, especially in precancerous cells where COX-2 is overexpressed. A downstream product of COX-2, PGE_2_, which often causes inflammation, can be modified, and become several short-lived so-called electrophilic oxo-derivative (EFOX) molecules that strongly regulate cell proliferation through the Nrf2/keap1/ARE pathway (Figure 2).

Figure 2 depicts a potential mechanism of COX-2 antioxidative activity via EFOX molecules, where reactive oxygen species (ROSs) are hypothesized to trigger antioxidative and anti-inflammatory effects. We recently demonstrated that UA, VEPS, APS and their combined application significantly reduced expression of COX-2 and lowered PGE_2_ concentration in HepG2 cells and inhibiting cellular proliferation. The mechanism for this inhibition may be due to the inhibition of COX-2, which is thought to increase oxidative stress because of the decreased expression of EFOX molecules that mediate gene expression of superoxide dismutase (SOD) and other AREs [65]. This is because the ARE family creates an antioxidative, anti-inflammatory protective environment to increase cell proliferation [66]. Increased malondialdehyde (MDA) concentration, a metabolic product of fatty acids, also indicates an oxidative environment. MDA expression is significantly reduced by UA. In contrast, inhibition of MDA and cell proliferation only occurs at high concentrations of VEPS and APS. Thus, it is possible that inhibition of HepG2 cell proliferation by UA, VEPS, APS and their combined application is attributable to inhibition of COX-2 and the associated decrease in SOD activity, which increases the oxidative environment and induces apoptosis.

These compounds, especially VEPS, may also have cancer-preventive roles* in vivo* via regulation of the immune system [67,68,69], as VEPS can activate macrophages and lymphocytes without causing severe inflammation or disease. Notably, some correlation between the dietary use of viili and cancer prevention has been suggested [70].

## 6. Anthocyanins

**Figure 3:**
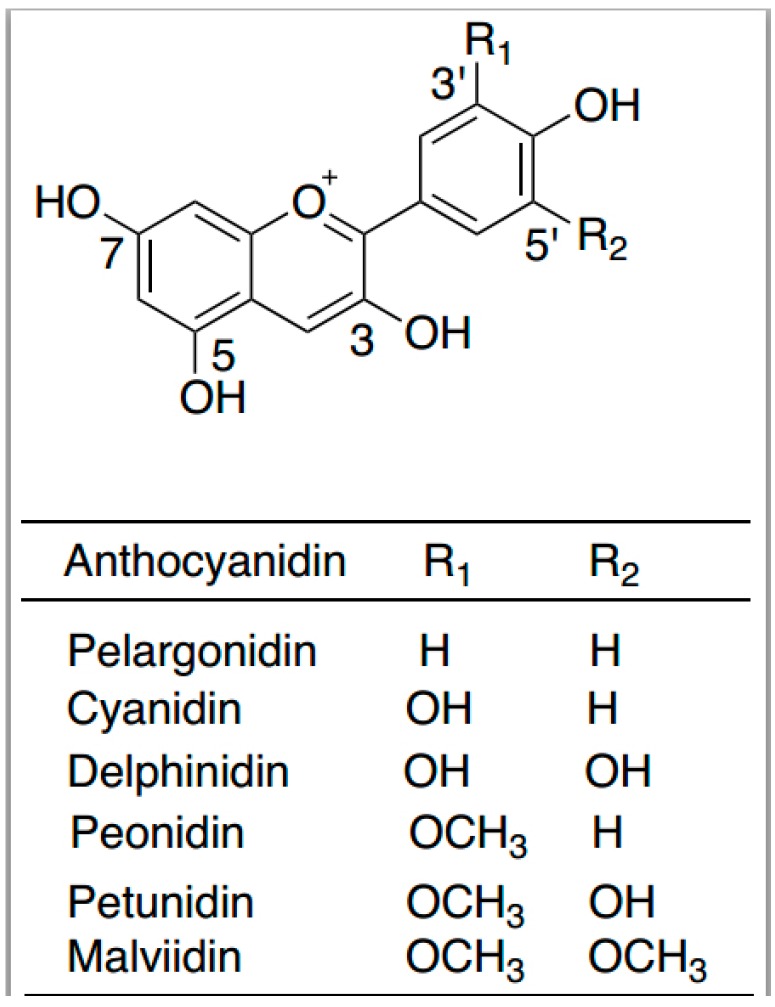
Structural formulas of common anthocyanidins.

Anthocyanins are water-soluble pigments derived from 2-phenylbenzopyrylium. They consist of an aglycone (anthocyanidins), sugars and/or acyl groups belonging to the flavonoid group characterized by a C6-C3-C6 skeleton. Major members include pelargonidin, cyanidin, peonidin, delphinidin, petunidin and malvidin (Figure 3), which have diverse patterns of glycosylation and acylation. Owing to the presence of flavylium cations (2-phenylbenzopyrylium), anthocyanins appear as red, blue or violet depending on their concentration, structure and environment [47].

In addition to their widely known use as natural food colorants, anthocyanins are also used as human nutritional supplements, as accumulating evidence has connected the intake of anthocyanin-rich foods with a reduced risk of chronic diseases such as cancer, cardiovascular diseases and Alzheimer’s. As these diseases may result from oxidative stress, it is understandable that the nutraceutical properties of anthocyanins are mainly attributed to their antioxidant activities including free radical scavenging, metal chelating and protein binding [71]. However, the relevance of their antioxidant activity is challenged by* in vivo* findings that circulating flavonoids have low concentrations (0.1–1 μM), and that antioxidant activity, especially the hydrogen-donating property of phenolic hydroxyl groups, can be impaired by conjugation with other molecules [72,73]. Therefore, more research is needed to understand the health benefits of plant anthocyanins and their potential chemopreventive mechanisms [74,75].

**Figure 4:**
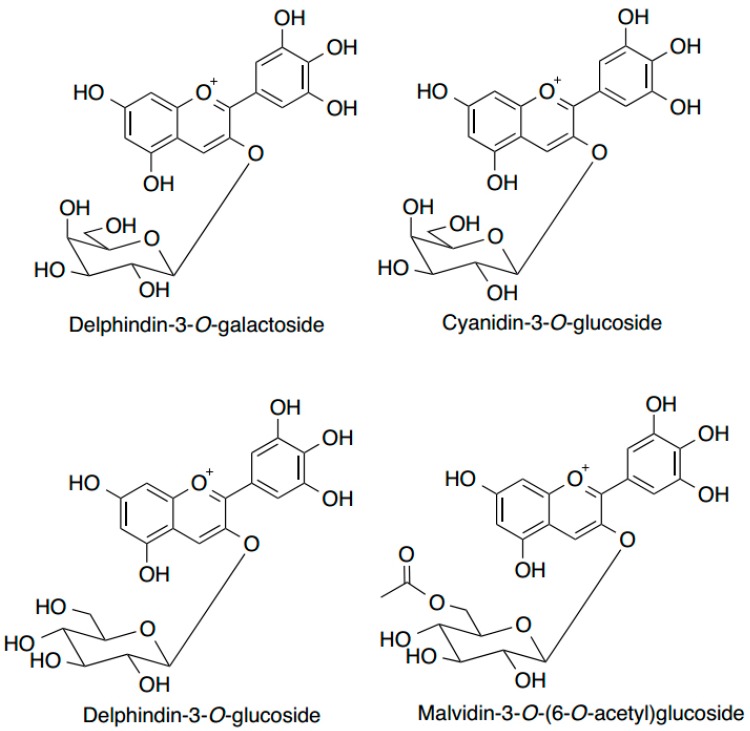
Examples of the major anthocyanins present in highbush blueberries (*Vaccinium corymbosum* L.). All examples have a sugar moiety at position 3.

Blueberries have enjoyed worldwide popularity owing to increasing awareness of their health benefits. We place particular emphasis on the highbush blueberry (*Vaccinium corymbosum* L.) in this review, because it is common to the northern hemisphere and the focus of our research. Blueberries are one of the richest sources of plant anthocyanins among common fruits and vegetables in terms of both variety and content [76,77]. Up to 25 anthocyanins have been identified in wildtype highbush blueberry, and glucosides of delphinidin, cyanidin and malvidin are the predominant fractions among these (Figure 4) [78]. This is in accordance with earlier findings [79,80]. The total anthocyanin content in highbush blueberries of different genotypes ranges from 25–495 mg/100 g fresh fruit [79]. Such high levels are strongly correlated (*r* = 0.90) with their antioxidant activity, which is three-fold higher than strawberries and raspberries as determined by the oxygen radical absorbing capacity (ORAC) assay [81]. Based on this same method, blueberry anthocyanins were found to contribute to over 50% of the total antioxidant activity, while ascorbate accounted for less than 10% of the total activity [76,82].

## 7. Anthocyanins and Modulation of DNA Repair

**Figure 5:**
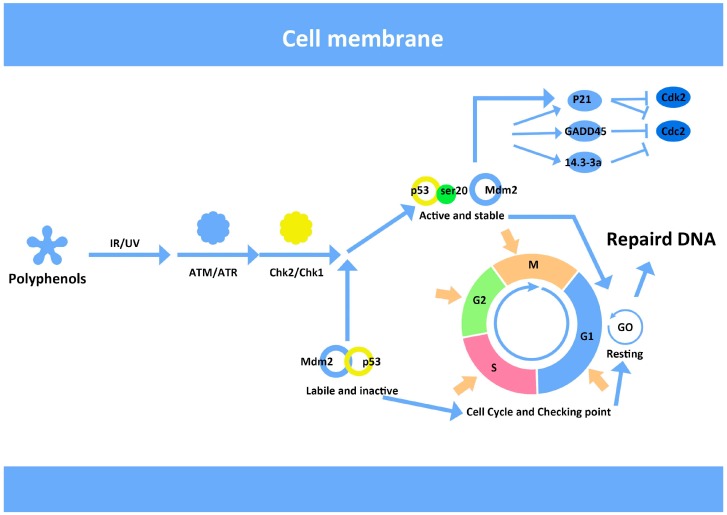
Hypothetical mechanism of DNA repair activation facilitated by blueberry anthocyanins (BA). Following DNA damage after ionizing or ultraviolet radiation (UV), the protein kinases ATM/ATR are activated, which modifies checkpoint-signaling pathways in the cell cycle. This is where BA possibly regulates cellular processes. The diagram is adapted from multiple illustrations based on publicly available materials, particularly a netbook [89].

Oxidative damage to DNA frequently leads to gene mutation and the potential initiation of carcinogenesis. Base repair in DNA damage is a simple process, but the repair of extensive DNA damage requires a complex set of molecular controls in mammalian cells. Cell cycle checkpoints in DNA replication and a complex network of sensor proteins ensure DNA fidelity [83,84]. In response to double-strand DNA breakage, deletion or fragmentation, the proteins ATM and H2AX are triggered and bind to unhelixed DNA between chromatin, where the complex of DNA repair machinery will then bind [85]. Signal transducers and pathways influenced during different parts of the cell cycle determine cell cycle arrest, DNA repair or apoptosis [86,87]. The fate of vertebrate somatic cells is decided in the G1 phase of the cell division cycle. We have reported that blueberry anthocyanins (BA) have a protective effect in UV-irradiated cells, which may be related to their antioxidant activity. Our findings demonstrated that DNA was significantly damaged after UV irradiation, with increased expression of p53 and p21 protein. In contrast, cells pre-treated with blueberry anthocyanin, had decreased p53 and p21 protein expression [88], thus indicating that BA can influence the DNA repair machinery (Figure 5).

Apoptosis induced by UV irradiation and its reversal by BA as observed in our study is associated with oxidation and antioxidants [90,91]. The oxidative environment created by the UV dose was not lethal even though typical apoptotic blebs were observed by scanning electron microscopy. Similarly, DNA fragmentation was observed by the comet assay, however the degree of fragmentation was relatively small compared with the whole nuclear genome. The effect of BA on gene and protein expression was also analyzed. Gadd45 and MDM2 were persistently overexpressed beyond 24 h; which made it possible for BA-assisted DNA repair to occur to reverse apoptosis.

It is widely known that p53 is closely associated with DNA damage and repair via the regulation of its downstream genes in reaction to DNA damage [92]. When DNA damage is induced by UV light or ionizing radiation, p53 activates the expression of genes, such as p21 and Gadd45. The expression of Gadd45 allows cells to arrest in the G1/S phase to induce DNA repair and regulates apoptosis signaling pathways and survival [93]. Meanwhile, MDM2 is activated by p53 and then phosphorylated and ubiquitinated by protein kinases to reduce inhibition of p53 by MDM2 [94]. Until the DNA damage is repaired, MDM2 is resynthesized to inhibit and degrade p53 protein and to promote cell cycle to return to the normal condition (Figure 5).

We confirmed that HepG2 cells were arrested in the G1 phase at a dose of 30 mJ/cm^2^ UV irradiation. Gadd45 and MDM2 proteins were significantly increased 12 h after irradiation and reduced by pretreatment with BA. We speculate that the protective effect of blueberry anthocyanins on DNA damage by UV irradiation is owing to its antioxidant activity, ability to scavenge free radicals and the regulation of relevant DNA repair proteins.

## 8. Antioxidants Inhibit Migration and Invasion of Cancer Cells as Indicated by HIF-1 and S100A4 Expression *in Vitro*

It recent years, a greater understanding of the mechanisms regulating cell migration and invasion has been gained. Although* in vivo* conditions cannot be replicated perfectly* in vitro*, the molecular mechanisms should be largely similar. We previously used a cell wound scratch assay and Transwell migration assay [95] to observe cell migration and invasion* in vitro*, investigating HIF-1 and S100A4 activity respectively. HIF-1 is associated with cell migration as hypoxia is a growth-promoting factor that activates fermentation and migration factors. The HIF-1α protein is the primary regulator and its active subunits are strictly regulated by oxygen concentrations. As almost all solid tumors are hypoxic, the inhibition of HIF-1 involves a chemotherapy approach. A staurosporine derivative (UCN-01) was previously found to block HIF-1 in prostate cancer cells by promoter trans-activation, thereby blocking the formation of new blood vessels. Colchicine and vincristine also inhibit HIF-1. Moreover, three types of dietary lignans were shown to reduce the proliferation of human breast cancer T47D cells via the selective inhibition of HIF-1α [96].

According to recent findings, the proteasome pathway also accelerates tumor and HIF-1α protein degradation, followed by the down-regulation of *VEGF* mRNA transcription and protein secretion. These results thus revealed a new angiogenesis inhibition pathway [97]. Saponins are amphipathic glycosides, which are soap-like foaming agents with both hydrophilic and hydrophobic properties. They are widely distributed in several different types of plants and have many biological activities including anti-tumor, anti-inflammatory, immune regulation, anti-viral and anti-fungal properties. Some saponins, such as ginseng saponins and anax notoginseng saponins, have been shown to regulate HIF-1 expression. For example, ginseng saponins significantly enhanced HIF-1α protein expression in the mouse cerebral cortex under hypoxic conditions [98].

We previously used ginseng saponin and the saponin from the leaf of the herb *Gynostemma pentaphyllum* to conduct a wound scratch migration assay in HepG2 cells and observed inhibition of HIF-1α under hypoxic conditions (unpublished data). Similarly, the inhibition of invasion by coix polysaccharides was also demonstrated using a Transwell assay in hepatocarcinoma and non-small cell lung cancer cells (NSCLC). The inhibition of migration and invasion of human A549 NSCLC cells was correlated with the* in vitro* down-regulation of S100A4 [99].

**Figure 6:**
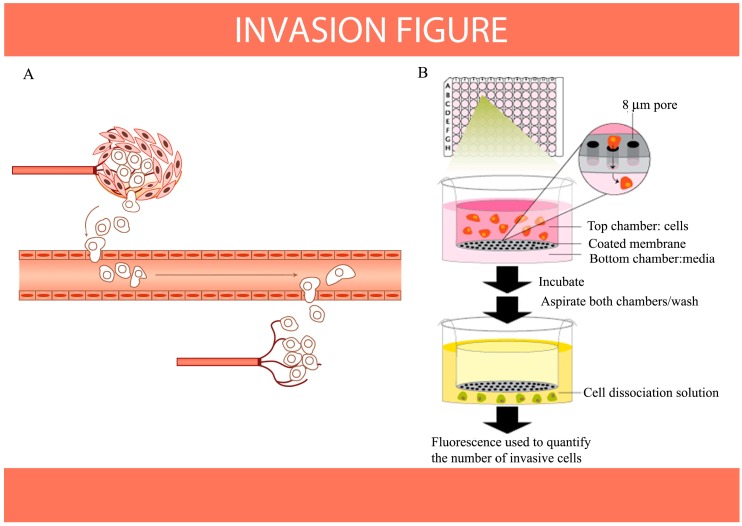
Aggressive malignant cancer cells are able to invade other tissues or organs with microvessels and can also induce angiogenesis. The progression from primary to metastatic tumor is a multigenic, multistep process that involves cell-cell and cell-extracellular matrix adhesion, tissue invasion and/or migration and angiogenesis. (**A**) Diagram of metastatic cancer invasion (adapted from [100] with permission from Nature Publishing Group, copyright 2005); (**B**) diagram of the* in vitro* invasion assay.

The mechanisms of cancer cell migration and invasion have been well studied. Cancer therapeutics designed to target adhesion receptors or proteases have proven not to be effective in slowing tumor progression in clinical trials. This may be owing to the fact that cancer cells can modify their migration or invasion mechanisms in response to changes in environmental conditions. However, as the processes of cell migration and invasion are integral to embryonic development and the functioning of organisms (Figure 6A), we have continued to investigate the mechanisms of inhibition of migration and invasion in cancer prevention and protection (Figure 6B). Cancer metastasis is coordinated by cell-cell communication and signaling, which may possibly be inhibited by dietary compounds, even though the details of the processes and mechanisms remain largely unknown.

## 9. Conclusions

**Figure 7:**
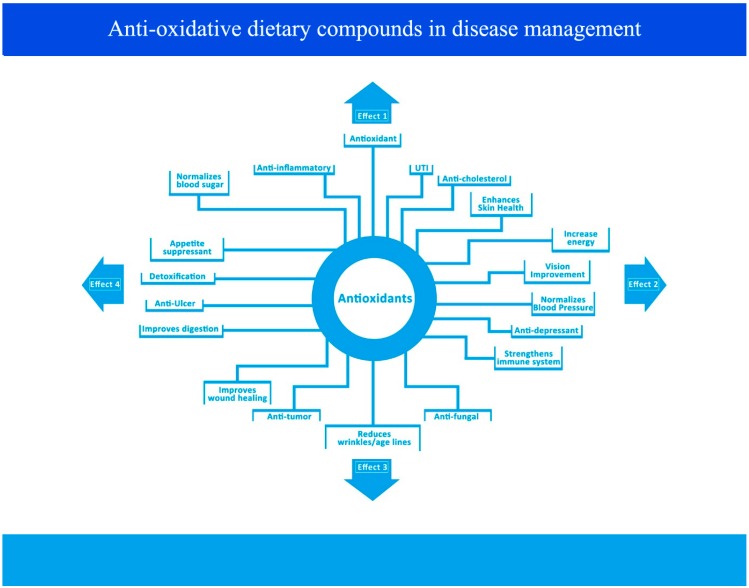
Commonly recognized functions of dietary antioxidants divided into four main types. Effect 1: Prevention or protection against inflammatory diseases such as urinary tract infections (UTI); Effect 2: Improvement of blood circulation, including cardiovascular diseases (CVD); Effect 3: Prevention of and protection against carcinogenesis; Effect 4: Improvement of the function of the gastric and intestinal systems.

A healthy diet is known to be beneficial in helping to prevent disease. Naturally derived polysaccharides from coix and viili, anthocyanins from blueberry, saponins from ginseng and ursolic acid are all similar to other antioxidants in that they are thought to contribute to good health via multiple mechanisms (Figure 7), including the modulation of gene expression. It is scientifically and culturally accepted that a balanced diet is able to lower the risk of disease. However, caution regarding the use of dietary antioxidants in health care and disease prevention remain, although there are clearly proven benefits. However, the multiple functions of dietary antioxidants frequently results in adverse effects, thus caution is needed in their application. For example, ginseng can alter blood glucose concentrations, therefore it is not advised for use in diabetic patients. As most mechanisms of these multiple functions of dietary antioxidants remain unclear, further studies are required.

The antioxidants we tested* in vitro* have been shown to significantly influence cell growth, DNA repair and mitochondrial membrane-mediated apoptosis. Although the cellular environment* in vivo* is much more complex, our studies suggest that the signals observed* in vitro* may provide clues for potential therapies in patients with chronic diseases, and also reveal the challenges facing application of dietary antioxidants.
